# Dexmedetomidine Alleviates Visceral Pain by Modulating a Pro-Inflammatory Macrophage-Associated Gene Network

**DOI:** 10.3390/biomedicines14081798

**Published:** 2026-08-10

**Authors:** Peng Ke, Yuying Li, Feng Liu, Liangcheng Qiu, Han Wu, Xiaodan Wu

**Affiliations:** 1 Department of Anesthesiology, Shengli Clinical Medical College of Fujian Medical University, Fuzhou University Affiliated Provincial Hospital, Fujian Provincial Hospital, Fuzhou 350001, China; kepeng@fzu.edu.cn (P.K.); sy1668@fzu.edu.cn (Y.L.); liufeng@fjmu.edu.cn (F.L.); 2023030328@fjmu.edu.cn (L.Q.); 2 Department of Anesthesiology, Fujian Medical University Union Hospital, Fuzhou 350004, China

**Keywords:** dexmedetomidine, molecular docking, macrophages, visceral pain

## Abstract

**Background/Objectives**: The clinical management of visceral pain remains a significant challenge. Our previous study confirmed the analgesic efficacy of dexmedetomidine (Dex) in a mouse model of inflammatory visceral pain (IVP). However, the underlying molecular mechanism, particularly regarding immune regulation, has not been fully elucidated. **Methods**: In the present study, transcriptomic profiling of both physiological and disease states was performed to characterize associated molecular and immune signatures. Key driver genes were identified by integrating differentially expressed genes (DEGs) from the IVP model with potential Dex targets derived from network pharmacology. Molecular docking simulations evaluated binding interactions. The correlations between Dex’s targets and immune cell infiltration patterns, with a focus on macrophages, were analyzed. **Results**: Multi-tissue analysis revealed a strong association between IVP pathogenesis and pro-inflammatory immune responses, specifically a shift in macrophage polarization. Within this dysregulated network, seven proteins were identified as direct potential targets of Dex. Among these, six core targets, such as ADGRF1, IDO1, JAK3 and NR1H4, demonstrated the most significant correlations with M1-like macrophages and exhibited strong in silico binding potential with Dex. **Conclusions**: This integrated analysis suggests that Dex may alleviate visceral pain by modulating a specific gene network linked to pro-inflammatory macrophage activation, thereby promoting the restoration of immune balance. Our findings provide a novel mechanism-informed perspective for the application of Dex in visceral pain therapy.

## 1. Introduction

IVP is a debilitating core symptom of gastrointestinal disorders such as inflammatory bowel disease and a major contributor to reduced quality of life [1]. Current analgesic strategies often provide insufficient relief or are limited by central nervous system side effects, highlighting a significant unmet clinical need [2]. This therapeutic challenge stems from an incomplete understanding of the disease’s complex molecular mechanisms [3], particularly the dynamic interplay between the nervous and immune systems [4].

The prevailing view has historically attributed visceral pain primarily to abnormal neuronal excitability [5]. However, contemporary research has established the immune system as a central player in pain initiation and maintenance [6]. This discovery of the neuro-immune axis has opened new therapeutic avenues beyond mere neuronal suppression [7]. Immune microenvironment dysregulation in key pain transmission sites, including the spinal cord and the gut, characterized notably by the polarization of macrophages towards a pro-inflammatory phenotype, is now recognized as a critical driver of pain sensitization [8].

Macrophages, as widely distributed leukocytes in the body, play a central role in pain and immune regulation through their phenotypic plasticity [9]. During acute inflammation, tissue-resident and infiltrating macrophages are not functionally homogeneous but rather exhibit a dynamic balance between the pro-inflammatory M1 phenotype and the anti-inflammatory M2 phenotype. This polarization state serves as a critical pivot determining the progression of inflammation and the outcome of pain [10]. Accumulating evidence indicates that an imbalance in macrophage polarization is a key mechanism driving pathological pain. In models of neuropathic pain and osteoarthritis, M1 macrophages have been shown to exacerbate neuroinflammation and sustain pain hypersensitivity by releasing pro-inflammatory cytokines such as TNF-α, IL-1β, and IL-6 [11]. In contrast, M2 macrophages exert endogenous analgesic and tissue-repair effects through the secretion of anti-inflammatory factors like IL-10 and TGF-β [12]. Therefore, M1 macrophages contribute to pain maintenance, while an increase in M2 macrophages can even reverse pain through direct contact with damaged neurons [13], clearly illustrating the cellular basis of “M1 promoting pain, M2 alleviating pain”.

Despite the established importance of immune regulation, translating this knowledge into effective treatments remains difficult [14]. A major obstacle is the absence of a complete molecular map of the key regulators governing the immune microenvironment in IVP. The identity of the genes that orchestrate the disease’s immune signature and their potential as precise drug targets are pressing unanswered questions. Dex, a sedative-analgesic agent commonly used in clinical practice, is characterized by its ability to induce a natural sleep-like sedative state, offering a compelling pharmacological profile [15]. As a highly selective α2 adrenergic receptor agonist, it demonstrates broad perioperative utility and exhibits sedative and analgesic potential throughout the perioperative period [16]. Our previous research has confirmed that Dex has a significant analgesic effect in IVP [17]. However, the underlying molecular mechanism remains unclear. Could the analgesic effect of Dex be partly mediated through its direct regulation of pain-associated immune gene networks? Confirming this would not only reveal a novel mechanism of action for this established drug but could also provide a roadmap for discovering next-generation precision analgesic targets.

Based on this premise, we hypothesize that Dex alleviates IVP by modulating a specific gene network linked to pro-inflammatory immune responses, particularly macrophage activation, thereby restoring immune homeostasis. To test this hypothesis, we integrated multi-dimensional omics data to delineate the disease-associated immunogenic landscape. Using network pharmacology and molecular docking, we systematically identified and validated its direct candidate targets. We aimed to construct a complete drug-target-immunity-analgesia axis. This study bridges the knowledge gap between pharmacology and immunology, offering a novel perspective on the mechanism of Dex and laying a theoretical foundation for developing immune-based analgesic strategies for visceral pain.

## 2. Materials and Methods

### 2.1. Animals and Methods

No additional animal experiments were conducted for this study. Therefore, new ethical approval was not required. Our previously published work [17] fully characterized the analgesic effects of Dex in the acetic acid-induced visceral pain model. Briefly, the IVP model was induced by intraperitoneal injection of 0.7% acetic acid (10 mL/kg) in male C57BL/6 mice. Dex was administered subcutaneously at 180 μg/kg immediately after baseline pain assessment, with a repeated dose on the following day. Behavioral assessments were performed by an investigator blinded to group allocation. For full methodological details, please refer to our previous report.

### 2.2. Data Acquisition and Preprocessing

Transcriptomic datasets relevant to IVP research were obtained from the GEO database [18]. Two independent datasets were included in the analysis. GSE131619 contains mouse dorsal root ganglia transcriptomic profiles from visceral and somatic sensory neurons, providing a reference for baseline molecular differences between visceral and somatic afferent pathways [19]. GSE11223 comprises colonic tissue transcriptomic data from patients with ulcerative colitis, a condition characterized by visceral hypersensitivity and chronic abdominal pain, and healthy controls, serving as an inflammatory reference relevant to the tissue microenvironment of IVP [20].

### 2.3. Identification of DEGs

DEGs were identified using NetworkAnalyst [21], a web-based platform that implements the Limma package. After standard quality control, gene expression profiles from visceral and somatic neurons were compared under physiological conditions to identify DEGs that may underlie differential visceral versus somatic sensation. To capture biologically meaningful changes while accounting for the subtle transcriptomic signature of somatic sensation, the thresholds were set at an absolute log2 fold change (|log_2_FC|) ≥ 1 and Benjamini–Hochberg adjusted *p*-value < 0.05. For the identification of DEGs under pathological conditions (IVP versus control), a similar analytical principle was applied using the GEO2R tool on the GEO database, with thresholds of |log_2_FC| ≥ 0.2 and Benjamini–Hochberg adjusted *p*-value < 0.05, as the expression changes in this dataset were relatively modest. Although the fold-change thresholds differed between datasets, both analyses applied the same Benjamini–Hochberg adjusted *p* < 0.05, ensuring consistent statistical stringency across datasets.

### 2.4. GSEA and Over-Representation Analysis

Functional enrichment analysis was performed using two complementary approaches. First, gene set enrichment analysis (GSEA) was conducted via WebGestalt [22] to identify coordinated changes across biological pathways, a method well suited for detecting subtle but consistent expression trends. This analysis followed the platform’s standard protocols for evaluating biological processes and signaling pathways.

In parallel, an over-representation analysis of Gene Ontology terms and Kyoto Encyclopedia of Genes and Genomes (KEGG) pathways was performed using Metascape [23]. This approach helped identify functional categories that were significantly enriched among the DEGs. In addition, the protein–protein interaction network derived from the analysis was used to pinpoint hub genes with high topological importance.

### 2.5. Estimation of Visceral Pain Related Immune Cell Infiltration

The relative proportions of immune cell types under physiological and pathological conditions were estimated using the CIBERSORT deconvolution algorithm, a well-validated computational method for inferring immune cell composition from bulk tissue gene expression profiles [24]. The analysis was applied to both the GSE131619 and GSE11223 datasets to quantify alterations in the immune microenvironment between normal states and states associated with visceral pain, leveraging the algorithm’s established reliability in characterizing immune cell dynamics.

### 2.6. Identification of the Potential Target of Dex

The molecular targets of small-molecule chemical drugs can be reliably predicted based on their chemical structures using established computational tools. To identify the potential targets of Dex, its SMILES identifier was first retrieved from PubChem [25]. This identifier was then independently queried against three target prediction databases: ChEMBL [26], SwissTargetPrediction [27], and TDR Targets [28]. The results from all three sources were consolidated to generate a comprehensive list of putative targets for Dex. Finally, the functional associations among these predicted targets were analyzed using the STRING database [29].

### 2.7. Molecular Docking of Dex with the Target Protein

The molecular docking verification procedure was performed as follows. The structural data of the primary active compound was downloaded from the PubChem database (https://pubchem.ncbi.nlm.nih.gov/) and saved in SDF format. The three-dimensional crystal structure of the target protein was retrieved from the PDB database (http://www.rcsb.org/) and saved in PDB format [30]. Using PyMOL software, water molecules and co-crystallized ligands were removed from the protein structure, and the GetBox Plugin was employed to define the binding-site parameters. Both the processed protein and the compound files were subsequently imported into AutoDock Tools 1.5.6 for conversion to PDBQT format. Molecular docking was then carried out using AutoDock Vina 1.1.2. Finally, the docking results were visualized with PyMOL 2.6.0.

### 2.8. Correlation Analysis Between Targets and Immune Cells

To investigate potential relationships between the target protein of Dex and immune cell composition, Pearson correlation analysis was conducted using R software. This analysis examined the association between the expression level of the target protein and the relative abundances of immune cell populations. The resulting correlation matrix was visualized as a heatmap using R’s statistical graphics capabilities to provide an intuitive representation of the interaction patterns.

### 2.9. Molecular Dynamics Simulation

All molecular dynamics (MD) simulations were performed using GROMACS 2022.2. The receptor topology was generated using the pdb2gmx tool with the Amber99SB-ILDN force field [31]. Ligand topologies and parameters were prepared using Sobtop_1.0 (dev3.1) based on the GAFF2 force field [32], and atomic partial charges were assigned using the RESP method. The complex was solvated in a cubic box with TIP3P water molecules, maintaining a minimum distance of 1.0 nm between the protein and the box edges. Na^+^ and Cl^−^ ions were added to a final concentration of 0.15 M using gmx genion to neutralize the system.

Long-range electrostatic interactions were treated with the Particle Mesh Ewald method, while van der Waals and short-range electrostatic cutoffs were set to 1.2 nm. All bonds were constrained using the LINCS algorithm, allowing an integration time step of 2 fs. Prior to production runs, energy minimization was performed using steepest descent followed by conjugate gradient methods. The system was then equilibrated under NVT (1 ns) and NPT (1 ns) ensembles with positional restraints on both protein and ligand. Temperature was maintained at 300 K using a V-rescale thermostat (τ = 1 ps), and pressure was maintained at 1 bar using a Parrinello–Rahman barostat (τ = 1 ns). After equilibration, unrestrained MD simulations were conducted.

Post-simulation analyses were performed using GROMACS built-in tools: RMSD (gmx rms) for backbone structural stability; RMSF (gmx rmsf) for residue-level flexibility; radius of gyration (Rg) (gmx gyrate) for structural compactness; solvent-accessible surface area (SASA) (gmx sasa) for protein–solvent interactions; hydrogen bond counts (gmx hbond) between protein and ligand; and free energy landscapes (FEL) were constructed using principal component analysis (PCA) followed by gmx sham.

### 2.10. Statistical Analysis

Unless otherwise stated, normally distributed data are expressed as mean ± standard deviation. Differences between two groups were assessed using a two-tailed Student’s *t*-test. For comparisons among three or more groups, one-way ANOVA followed by Tukey–Kramer post hoc test was applied. A *p*-value < 0.05 was considered statistically significant, with significance levels denoted as * *p* < 0.05, ** *p* < 0.01, *** *p* < 0.001, and **** *p* < 0.0001. All statistical analyses were performed using GraphPad Prism 7.0, NetworkAnalyst, and R software (R version 4.5.1).

## 3. Results

### 3.1. Transcriptomic and Functional Characterization of Physiological Visceral Sensation

**Figure 1:**
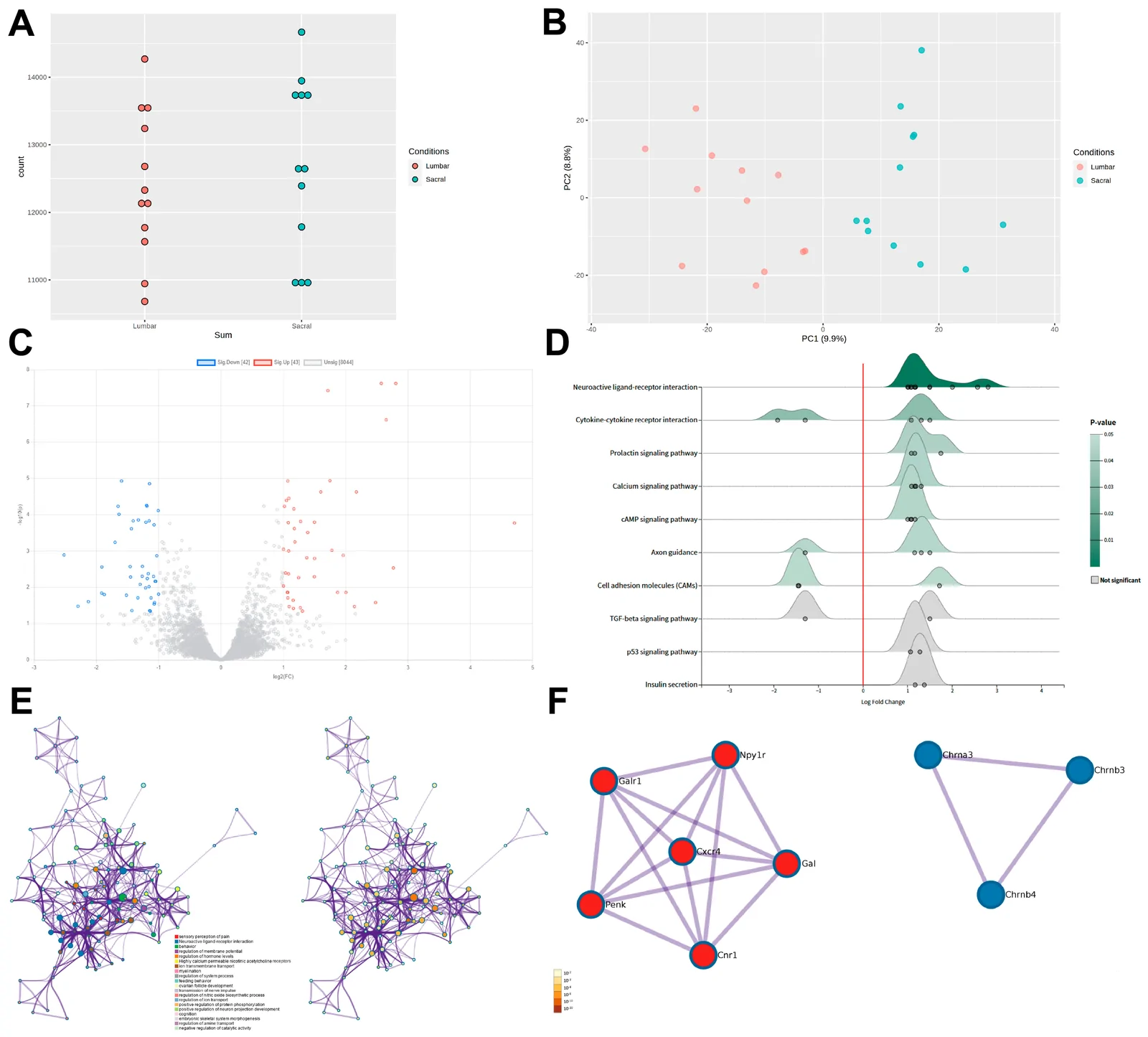
Transcriptomic and functional profiling of physiological visceral sensation. (**A**) Sample distribution and density in visceral versus somatic sensory tissues. (**B**) Principal component analysis plot illustrating transcriptional separation between visceral and somatic sensory tissue samples. (**C**) Volcano plot of DEGs comparing visceral to somatic sensory tissues. (**D**) Ridge plot from GSEA of the visceral sensory gene signature. (**E**) Functional enrichment network of biological processes associated with genes differentially expressed in visceral sensation. (**F**) Core protein-protein interaction module, identified by MCODE, from the network of visceral sensory signature genes.

Our previous study confirmed the analgesic efficacy of Dex in the IVP model [17]. To characterize the molecular landscape of visceral sensation under physiological conditions, we performed transcriptomic profiling of visceral and somatic sensory tissues. Analysis showed a clear separation in sample distribution and transcriptional profiles between the two tissue types (Figure 1A,B). Differential gene expression analysis identified 82 unique genes in visceral sensation (Figure 1C). GSEA of this visceral sensory signature revealed its significant association with several key biological pathways, including neuroactive ligand–receptor interaction, cytokine–cytokine receptor interaction, calcium signaling pathway, and cell adhesion molecules (Figure 1D). Functional annotation demonstrated that the DEGs in visceral sensory pathways were significantly enriched in specific biological processes, including sensory perception of pain, neuroactive ligand–receptor interaction, behavior, and regulation of membrane potential (Figure 1E). Key genes, including *Galr1*, *Npy1r*, *Cxcr4*, *Gal*, *Cnr1*, *Penk*, *Chrna3*, *Chrnb3*, and *Chrnb4*, were identified within the protein–protein interaction network using the MCODE algorithm, highlighting their association with visceral sensation (Figure 1F). Collectively, these results suggest that visceral sensory pathways are associated with a distinct molecular profile that may involve the immune-interactive molecular environment even under a non-pathological state.

### 3.2. Molecular Characterization of IVP and Immune Pathway Activation

**Figure 2:**
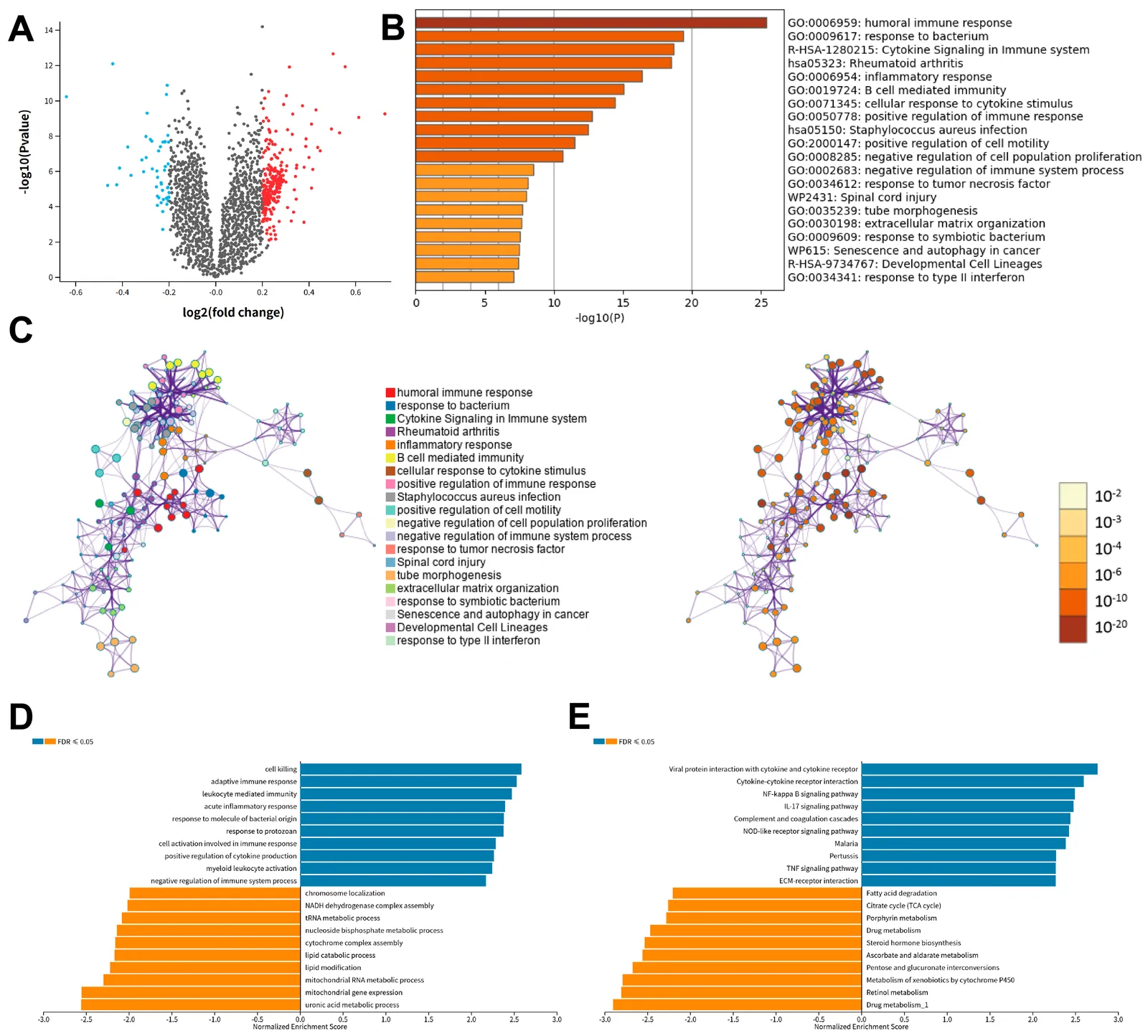
Transcriptomic alterations and pathway enrichment in IVP. (**A**) Volcano plot of DEGs in the IVP model versus control animals. (**B**) Functional enrichment analysis of biological processes among genes differentially expressed in the IVP model. (**C**) Network visualization of enriched biological processes associated with DEGs in the IVP model. (**D**) GSEA enrichment plot for a biological process gene set altered in the IVP model. (**E**) GSEA enrichment plot for a signaling pathway gene set enriched in the IVP model.

In the disease state, transcriptomic analysis of the IVP model identified a substantial number of DEGs (Figure 2A). Gene Ontology analysis revealed that these genes were significantly enriched in biological functions including humoral immune response, cytokine signaling in the immune system, inflammatory response, B cell-mediated immunity, negative regulation of immune system process, and spinal cord injury (Figure 2B), with a clear network of interactions existing among these functions (Figure 2C). Further analysis using GSEA indicated significant associations with biological processes such as adaptive immune response and cell killing (Figure 2D), as well as signaling pathways including cytokine–cytokine receptor interaction, leukocyte-mediated immunity, NF-κB signaling pathway, and IL-17 signaling pathway (Figure 2E). This comprehensive profiling confirms that the pathogenesis of IVP is strongly linked to a broad and interconnected activation of both innate and adaptive immune pathways.

### 3.3. Immune Cell Infiltration Dynamics in Visceral Pain Pathogenesis

**Figure 3:**
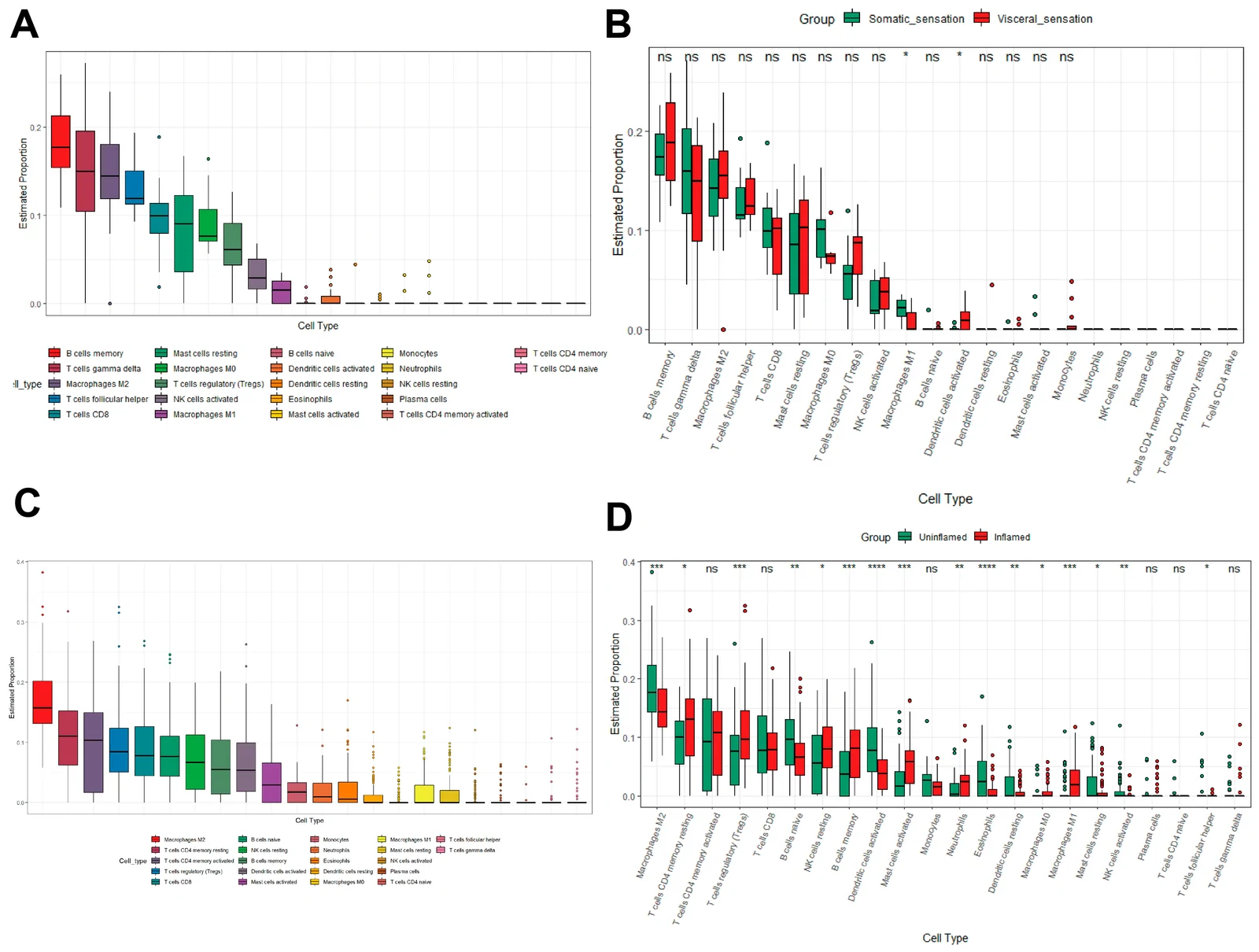
Characterization of immune cell composition in visceral sensory tissue under physiological and pathological conditions. (**A**) Relative proportions of major immune cell types in visceral sensory tissue under physiological conditions, as estimated by CIBERSORT analysis. (**B**) Comparative analysis of immune cell proportions between visceral and somatic sensory tissues under physiological conditions. (**C**) Relative proportions of major immune cell types in IVP model. (**D**) Comparative analysis of immune cell proportions between the IVP model and control. * *p* < 0.05, ** *p* < 0.01, *** *p* < 0.001, **** *p* < 0.0001; ns, not significant.

To explore the translational relevance of transcriptomic alterations at the cellular level, we analyzed the immune cell composition using CIBERSORT. Under physiological conditions, visceral sensory tissue exhibited a distinct immune profile compared to somatic sensory tissue (Figure 3A), characterized by a lower abundance of M1 macrophages and a higher proportion of activated dendritic cells (Figure 3B). In the IVP model, the immune cell composition differed from that of control tissues (Figure 3C,D). Specifically, the IVP model showed an increased proportion of M1 macrophages and decreased proportion of activated dendritic cells. Changes were also observed in other immune cell types, including M0 and M2 macrophages. Collectively, these results suggest an alteration in the local immune cell composition that may be associated with pain signaling.

### 3.4. Identification of Core Target Genes Linking Dex to Visceral Pain Immunity

**Figure 4:**
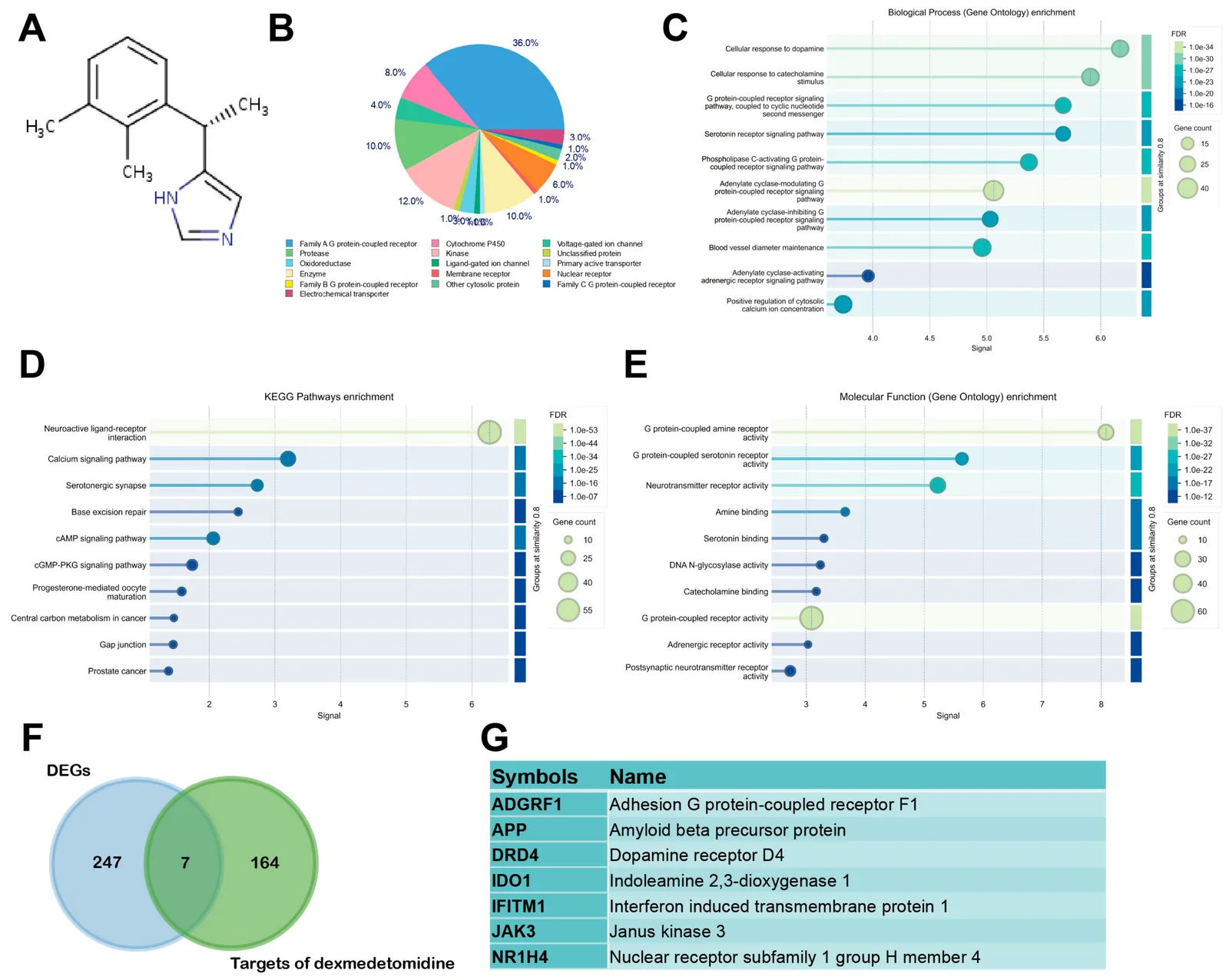
Identification of Dex targets and their overlap with visceral pain-related genes. (**A**) Chemical structure of Dex. (**B**) Classification of predicted molecular target types for Dex. (**C**) Enriched biological processes, (**D**) signaling pathways, (**E**) and molecular functions among all predicted Dex targets. (**F**) Venn diagram showing the overlapping genes between predicted Dex targets and genes with altered expression in the IVP model. (**G**) Expression patterns of the seven overlapping genes identified from the intersection analysis.

To elucidate the molecular mechanism through which DEX might interact with this dysregulated system, we conducted an integrative pharmacology analysis. The chemical structure of DEX is presented in Figure 4A. A comprehensive prediction yielded a total of 171 potential target proteins for DEX, with some of their molecular classifications detailed in Figure 4B. Functional enrichment analysis of these predicted targets revealed their significant association with specific biological processes, including cellular response to dopamine and catecholamine stimulus (Figure 4C), key signaling pathways such as calcium signaling and neuroactive ligand–receptor interaction (Figure 4D), and molecular functions like G protein-coupled receptor activity (Figure 4E). This profile aligns with the known pharmacological effects of DEX. To identify potential direct mediators, we intersected the predicted DEX targets with genes differentially expressed in the IVP model, resulting in seven overlapping candidate genes (Figure 4F). The basic information on these seven overlapping molecules is presented in Figure 4G.

### 3.5. In Silico Validation of Binding Affinity Between Dex and Candidate Targets

**Figure 5:**
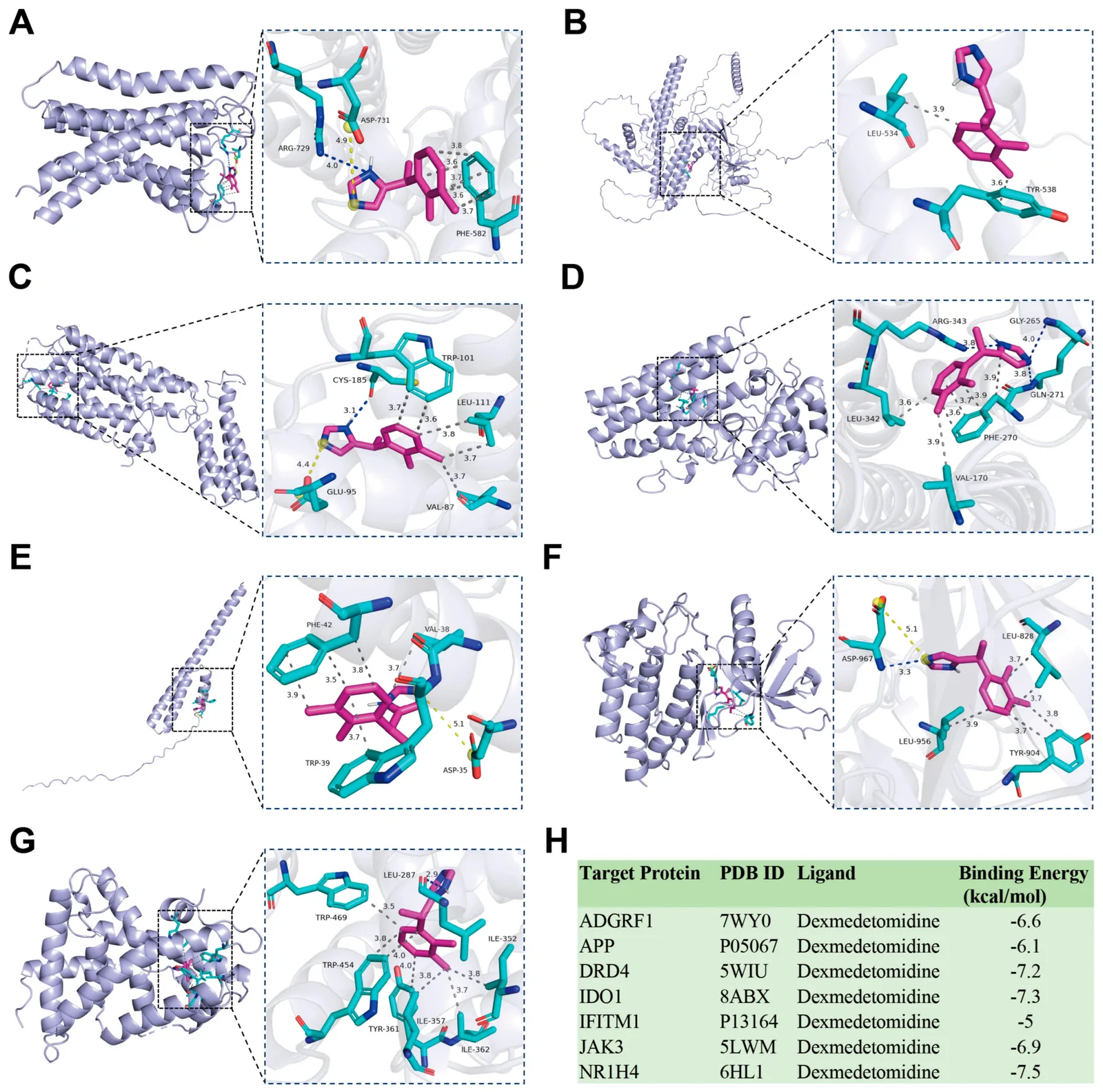
In silico validation of binding interactions between Dex and prioritized targets. Predicted binding modes and affinity scores for Dex docked to the protein structures of (**A**) ADGRF1, (**B**) APP, (**C**) DRD4, (**D**) IDO1, (**E**) IFITM1, (**F**) JAK3, (**G**) NR1H4. (**H**) Detailed molecular docking information.

Molecular docking simulations were performed to assess the structural feasibility of interaction between Dex and the protein products of the seven candidate target genes identified from the intersection analysis. The results predicted stable binding between DEX and all seven targets (Figure 5A–G). The calculated binding energies ranged from −5.0 to −7.5 kcal/mol as detailed and the accompanying table (Figure 5H). Notably, three targets, NR1H4 with a binding energy of −7.5 kcal/mol, IDO1 at −7.3 kcal/mol, and DRD4 at −7.2 kcal/mol, exhibited the strongest binding affinities. The remaining targets showed moderate to weaker binding, with JAK3 at −6.9 kcal/mol, ADGRF1 at −6.6 kcal/mol, APP at −6.1 kcal/mol, and IFITM1 at −5.0 kcal/mol. This spectrum of binding energies supports the plausibility of these proteins as potential direct molecular targets of Dex, with NR1H4, IDO1, and DRD4 representing the most energetically favorable interactions.

### 3.6. Correlation of Core Target Genes with Pro-Inflammatory Immune Infiltration

**Figure 6:**
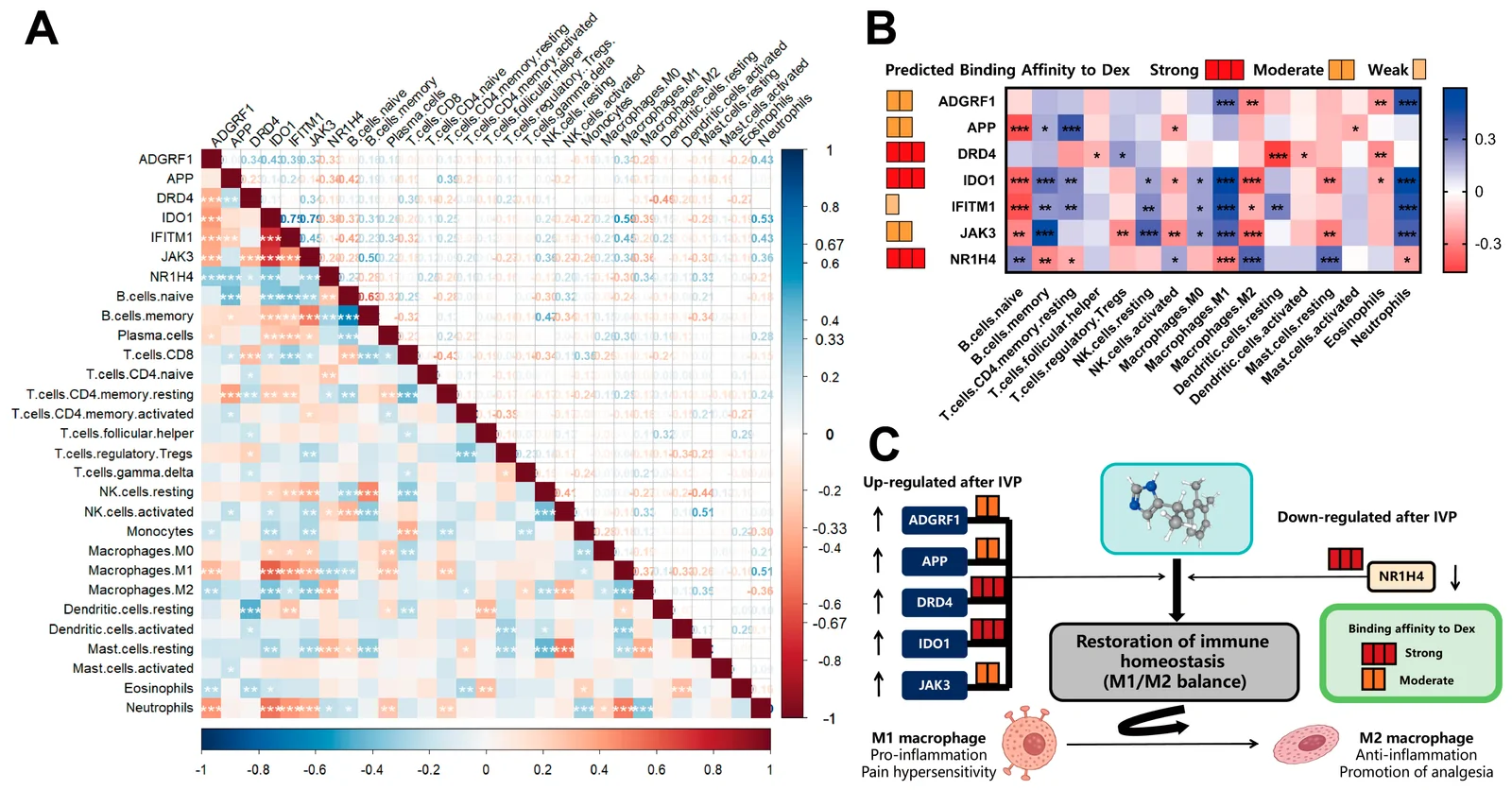
Correlation of Dex’s targets with specific immune cell populations in the IVP model. (**A**) Heatmap of Pearson correlation coefficients between the expression levels of seven targets and the relative abundances of infiltrating immune cells. (**B**) Detailed targets and immune infiltration situation. (**C**) Mechanism network diagram of Dex. * *p* < 0.05, ** *p* < 0.01, *** *p* < 0.001.

To elucidate the immunomodulatory role of Dex in pain, we analyzed the correlation between its core target proteins and immune cell infiltration in our model. Distinct associations were observed between the candidate target proteins and specific immune cell populations (Figure 6A). Further analysis revealed that the seven core proteins were significantly correlated with B cell and NK cell activation status, as well as macrophage polarization (Figure 6B). Among them, ADGRF1, IDO1, IFITM1, and JAK3 showed a clear tendency toward positive correlations with pro-inflammatory, pain-promoting M1 macrophages and negative correlations with anti-inflammatory, analgesia-associated M2 macrophages. In contrast, NR1H4 exhibited the opposite pattern, being negatively correlated with M1 macrophages and positively correlated with M2 macrophages.

Based on these findings and molecular docking results, we propose a regulatory network through which Dex may exert its effects (Figure 6C). Specifically, Dex may bind to ADGRF1, IDO1, APP, DRD4, and JAK3, inhibiting their activity and thereby reducing pro-inflammatory signaling. Conversely, its interaction with NR1H4 appears to stabilize this target protein, enhancing its protective and anti-inflammatory functions. Collectively, these results position the identified proteins as key modulators within the immune network of visceral pain, and suggest that Dex exerts dual regulatory roles by simultaneously suppressing pro-inflammatory pathways and promoting protective responses.

### 3.7. Molecular Dynamics Simulation Analysis

**Figure 7:**
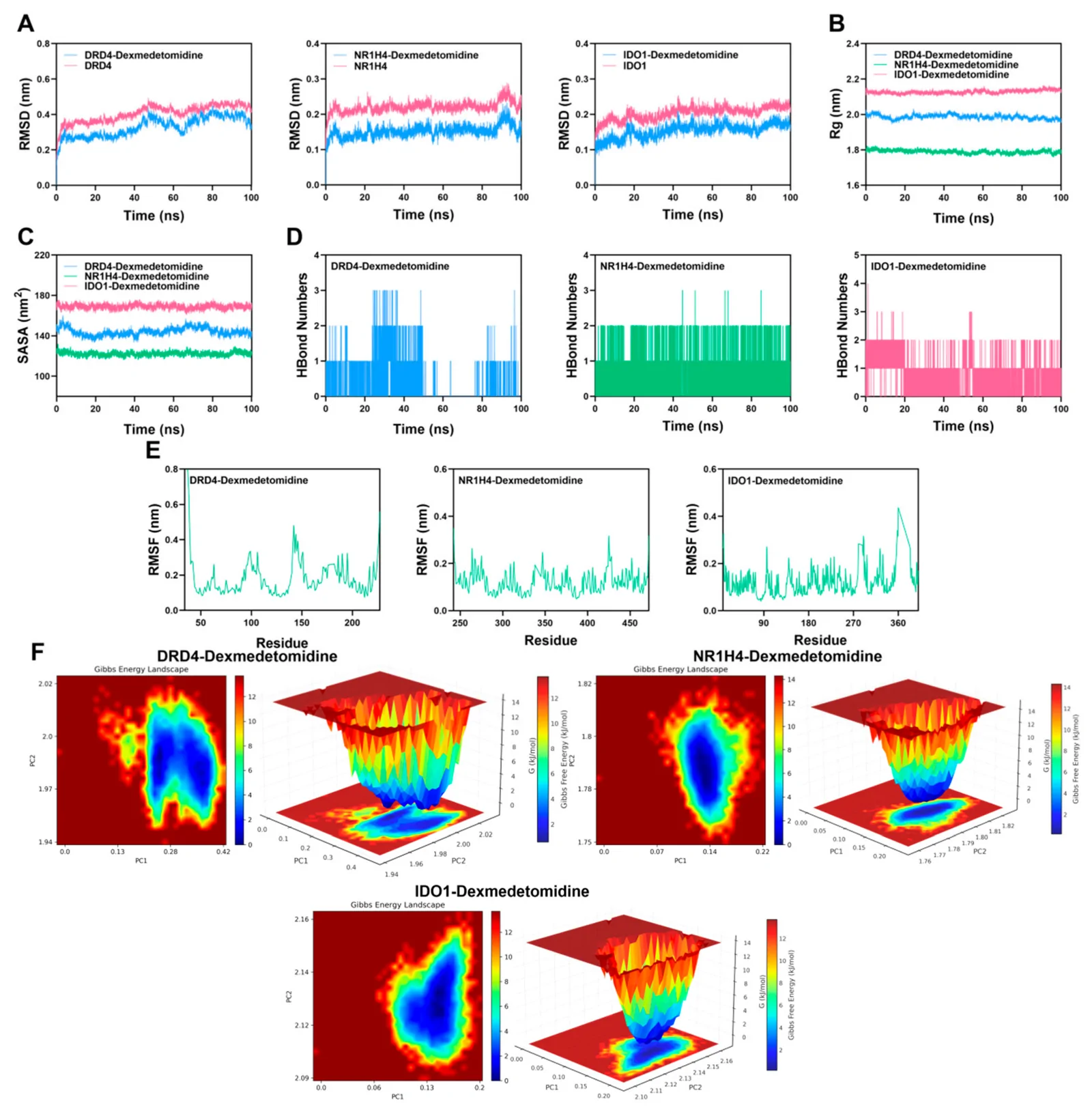
Molecular dynamics simulations of Dex with its potential targets (DRD4, NR1H4, and IDO1). (**A**) RMSD plots over 100 ns for each complex, confirming system equilibration and structural stability. (**B**) Radius of gyration (Rg), (**C**) SASA, and (**D**) intermolecular hydrogen bond (H-bond) counts over time, indicating maintained protein compactness, stable solvent exposure, and persistent protein–ligand interactions, respectively. (**E**) Per-residue RMSF for each complex, with key binding pocket residues highlighted by arrows. (**F**) FEL, each revealing a stable global energy minimum corresponding to the respective binding conformation.

Molecular dynamics simulation confirmed stable binding of dexmedetomidine to DRD4, NR1H4, and IDO1. All three protein–ligand complexes reached equilibrium within 100 ns, with RMSD values stabilizing at 0.40–0.43 nm (DRD4), <0.17 nm (NR1H4), and <0.21 nm (IDO1) (Figure 7A). Rg and SASA analyses revealed maintained structural compactness and stable solvent exposure for all complexes (Figure 7B,C). Intermolecular hydrogen bonds were consistently observed, with averages of approximately 1 (DRD4), 2 (NR1H4), and 2 (IDO1) bonds per trajectory frame (Figure 7D). RMSF profiles showed low residue flexibility in all three complexes (Figure 7E). Free energy landscape analysis identified single deep energy basins for each complex, corresponding to their most stable binding conformations (Figure 7F). Collectively, these results confirm that dexmedetomidine forms structurally stable complexes with DRD4, NR1H4, and IDO1, supporting their roles as direct molecular targets.

## 4. Discussion

This study provides an integrated exploration of a potential novel mechanism through which Dex alleviates IVP, combining behavioral pharmacology, multi-omics analysis, and computational modeling. Our findings delineate a coherent signaling axis linking the drug to specific molecular targets, immune cell infiltration, and ultimately pain behavior, offering a perspective that extends beyond its conventional neuromodulatory actions.

We first confirmed the reliable analgesic efficacy of Dex in our animal model [17], which aligns with extensive clinical evidence [33,34]. Importantly, Dex has been consistently shown to exert analgesic effects in both acute and chronic inflammatory pain models, with its efficacy further validated in experimental models of inflammatory bowel disease. Although inflammatory bowel disease datasets do not directly model pain behavior, they provide a clinically relevant human inflammatory reference that reflects the tissue microenvironment from which visceral pain emerges. Therefore, we reasoned that transcriptomic profiling of inflammatory bowel disease tissues could serve as a valuable resource for identifying inflammation-associated molecular pathways that may also contribute to IVP pathogenesis. In this context, our analysis of GSE11223 was not intended to substitute for an IVP-specific pain model, but rather to complement the murine behavioral data by offering a human-relevant inflammatory transcriptomic landscape for target exploration. Therefore, we reasoned that the immune microenvironment of inflammatory bowel disease tissues may serve as a relevant context for exploring Dex’s potential mechanisms in inflammatory visceral pain. More significantly, our transcriptomic data provide systematic molecular support for the concept of visceral pain as an immune-associated disorder. We found that visceral sensory pathways exhibit a distinct immune-related gene expression signature even under physiological conditions, suggesting an inherent state of immune readiness. For instance, CXCR4, as the receptor for SDF1, can recruit and activate immune cells such as macrophages and T cells to infiltrate inflammatory sites in the dorsal root ganglia or intestinal mucosa, thereby modulating neuronal excitability and pain signal transmission through the regulation of inflammatory cytokine release [35]. In the pathological state, a broad activation of inflammatory and immune pathways formed the core of the disease’s molecular signature. This observation strongly resonates with the prevailing view that neuro-immune interactions are central to visceral pain pathogenesis [36]. Our data refine this understanding by pinpointing a shift in macrophage polarization, specifically an increase in the pro-inflammatory M1 phenotype, as a key cellular correlate of the dysregulated immune microenvironment.

The central advance of this study lies in moving beyond phenomenological association to mechanism. By integrating network pharmacology with differential genomics, we identified seven candidate targets potentially responsible for the immunomodulatory effects of Dex. To further elucidate the immunoregulatory role of Dex in pain, we examined the correlation between these core target proteins and immune cell infiltration. Our results showed that these proteins were significantly associated with specific immune cell populations. Specifically, the seven core proteins exhibited notable correlations with the activation states of B cells and NK cells, as well as the polarization of macrophages. Among them, ADGRF1, IDO1, IFITM1, and JAK3 showed positive correlations with pro-inflammatory, pain-associated M1 macrophages and negative correlations with anti-inflammatory, analgesia-related M2 macrophages. In contrast, NR1H4 displayed an opposite correlation pattern.

Building on these findings and molecular docking results, we constructed a potential regulatory network underlying Dex’s action. Dex likely binds to ADGRF1, IDO1, APP, DRD4, and JAK3, inhibiting their activity and thereby reducing pro-inflammatory signaling. Conversely, its interaction with NR1H4 may stabilize this target protein, enhancing its protective and anti-inflammatory functions. Together, these results provide substantial evidence that Dex may exert dual regulatory effects by simultaneously suppressing pro-inflammatory pathways and promoting protective responses. This finding is significant because it situates a systemically acting drug within a disease-specific molecular network that varies with key immune phenotypes, offering a plausible mechanistic explanation for its selective effects.

Previous studies provide strong support for certain aspects of our findings. For example, genome-wide association studies and proteome-wide association studies have linked ADGRF1 to chronic pain in multiple body regions. Clinical evidence further confirms its relevance in treating conditions such as neck and shoulder pain [37]. The impact of the DRD4 gene variant on substance use in adulthood is predominantly mediated by the experience of chronic pain [38]. More direct evidence reported that knockout of the IDO1 gene in mice alleviates pain sensitization in models of chronic migraine [39]. The finding that morphine and ketamine alleviate cervical cancer pain and suppress JAK3 is consistent with the mode of action of Dex, which also appears to exert analgesia through JAK3 inhibition [40].

Synthesizing these findings, we propose a working hypothesis for the mechanism of Dex. While its analgesic effect has traditionally been attributed to the activation of central α2-adrenergic receptors and subsequent inhibition of neuronal excitability [41], our findings suggest an additional peripheral immunomodulatory pathway that may complement this central mechanism. We postulate that Dex may bind to and modulate proteins including ADGRF1, APP, DRD4, IDO1, JAK3 and NR1H4, potentially interfering with their roles in promoting M1 macrophage activation or sustaining a pro-inflammatory state. Notably, our observed correlation between DRD4 and M1-like macrophages in the IVP model is consistent with previous findings in a mouse cancer model, where dopamine activation of DRD4 reduced cAMP levels and suppressed M2 polarization of tumor-associated macrophages [42]. Similarly, other targets identified in our study have been linked to macrophage regulation in distinct pathological conditions. For instance, in a mouse model of liver fibrosis, corilagin was shown to alleviate fibrosis and suppress IDO1 expression. In vitro experiments further demonstrated that IDO1 inhibition was associated with macrophage polarization from the M1 to the M2 phenotype [43]. JAK3 phosphorylation has been implicated in inflammatory cytokine transcription in macrophages [44], and NR1H4 agonists exert anti-inflammatory effects by targeting peripheral immune cells including macrophages in models of experimental autoimmune encephalomyelitis and multiple sclerosis [45]. Collectively, these observations suggest that the immunomodulatory targets identified in our study may contribute to the analgesic effect of Dex.

To further validate the binding interactions predicted by molecular docking, we performed 100 ns molecular dynamics simulations for each of the three identified targets (DRD4, NR1H4, and IDO1) with dexmedetomidine. MD simulations provide a more physiologically relevant assessment of protein–ligand interactions by accounting for protein flexibility and solvent effects, which are not captured by static docking approaches. Our MD results consistently demonstrated that Dex forms structurally stable complexes with all three targets, as evidenced by equilibrated RMSD profiles, maintained structural compactness (Rg), stable solvent accessibility (SASA), persistent hydrogen-bonding interactions, and well-defined low-energy conformational basins (FEL). These findings provide strong computational evidence supporting Dex’s potential to directly engage these targets in IVP. However, we acknowledge that our findings are correlative and computational in nature. Further functional studies, such as cell-specific gene knockout or pharmacological blockade, are required to establish the causal relationship between these targets and Dex-induced pain relief.

Such intervention at the molecular and cellular level could ultimately help reverse the local immune imbalance, shifting the microenvironment from a pro-inflammatory state back towards homeostasis, thereby alleviating pain sensitization. This hypothesis offers a conceptual bridge connecting the drug’s established pharmacological profile to the emerging immunology of pain, framing analgesia as a potential consequence of restored immune homeostasis.

The strength of this study lies in its multi-tiered and complementary strategy, forming a consistent evidence chain from whole-animal behavior to systems biology and in silico validation. As a primarily computational and predictive investigation, however, these conclusions warrant cautious interpretation. The main limitation is that all functional inferences regarding “modulation” or “inhibition” are derived from correlative analyses and computational predictions, necessitating direct validation through subsequent experimental biology. Crucially, future work should involve knockdown or overexpression of genes like IDO1 and NR1H4 in cellular or animal models to observe the functional impact on macrophage polarization and pain behavior. Furthermore, clarifying whether Dex functionally activates or inhibits these targets will require confirmation through biochemical and cellular assays. We also acknowledge potential biases in cross-species and cross-tissue analyses. Nonetheless, the convergence of both datasets on overlapping macrophage-related inflammatory pathways, despite their biological disparities, reinforces the robustness of our findings. Lastly, future cross-validation using orthogonal deconvolution tools such as xCell, MCP-counter, or EPIC represents a logical next step to further consolidate our conclusions.

In conclusion, this study presents the first systematic blueprint suggesting that Dex may alleviate IVP by modulating a specific protein network closely associated with M1 macrophages. This work not only adds a new mechanistic dimension to this established drug, potentially repositioning it as an immunomodulatory analgesic, but also identifies target proteins such as DRD4, IDO1 and NR1H4 as promising candidates for developing next-generation analgesics with immunomodulatory functions. Future research that experimentally validates the role of these targets could advance the understanding of visceral pain treatment, shifting the focus from neuronal suppression toward the modulation of immune homeostasis.

## 5. Conclusions

This study proposes that Dex may alleviate visceral pain by modulating a specific immune-associated gene network, including key regulators such as DRD4 and IDO1, which are closely linked to pro-inflammatory macrophage activity. These findings suggest a novel, immunomodulatory mechanism of action for this established drug, extending beyond its traditional neurological effects. The identified targets provide a promising foundation for the future development of precision therapies for visceral pain.

## Data Availability

Data are available on request.

## Abbreviations

The following abbreviations are used in this manuscript:

Dex	Dexmedetomidine
IVP	Inflammatory visceral pain
DEGs	Differentially expressed genes
GSEA	Gene set enrichment analysis
KEGG	Kyoto Encyclopedia of Genes and Genomes
MD	Molecular dynamics
FEL	Free energy landscapes
SASA	Solvent-accessible surface area
